# Impact of anal precancer screening on patient-reported outcomes among men-who-have-sex-with-men living with HIV: a scoping review

**DOI:** 10.1097/CEJ.0000000000001001

**Published:** 2025-12-17

**Authors:** Emmi Suonpera, Yomna Gharib, Deirdre Sally, Shema Tariq, Richard Gilson

**Affiliations:** a Centre for Clinical Research in Infection and Sexual Health, Institute for Global Health, University College London; b Central and North West London NHS Foundation Trust, London, UK

**Keywords:** anal precancer, screening, HIV, men-who-have-sex-with-men, patient-reported outcomes

## Abstract

Interest in human papillomavirus (HPV)-related anal cancer screening among high-risk groups like men-who-have-sex-with-men living with HIV (MSMLWH) is high. Yet, the psychosocial impact of screening is not yet clear. We reviewed literature on patient-reported outcomes (PROs) associated with anal precancer screening among MSMLWH to identify current research priorities. In this scoping review, inclusion criteria were peer-reviewed studies of any type published in English since 2000 reporting PROs after anal precancer screening in MSMLWH. A database search (MEDLINE, EMBASE, and APA PsycINFO) was conducted in December 2024. Data were extracted independently by two authors using a standardised form. Eleven papers (nine cohorts) met the inclusion criteria, mostly from high-income countries. Participants were generally over 40 and had lived with HIV for greater than or equal to 10 years. All were involved in anal precancer screening studies; none were part of routine screening programmes. Ethnicity data were inconsistently reported. Papers covered four PRO domains: health-related quality of life (HRQoL), psychological impact, experience of screening procedures, and physical health. Screening was well-tolerated with minimal psychological or HRQoL impact. Negative impact related to screening procedures did not persist beyond receiving results. Low levels of pain and discomfort (≤11%) were reported. Some reported increased cancer-related worry, especially when further examination was needed. Systematic collection of PROs in this context remains uncommon. Existing evidence suggests screening is generally well tolerated with minimal psychological burden, though some studies note psychosocial effects. Using validated PRO measures can guide targeted support and inform the development of screening programmes that minimise psychological adverse effects.

## Introduction

Human papillomavirus (HPV)-related anal cancer disproportionately affects men-who-have-sex-with-men living with HIV (MSMLWH) because of the potential of HIV-induced immunosuppression and consequent continued HPV persistence. HIV coinfection enhances the carcinogenicity of HPV (Uuskula *et al*., 2025). Among men-who-have-sex-with-men (MSM), the prevalence of HPV infection remains high even at older ages in comparison with heterosexual individuals (Wei *et al*., 2021). Despite increased interest in gender-neutral vaccination, MSM still benefit less from herd immunity because of previous predominantly female-targeted HPV vaccination strategies (Lin *et al*., 2017; Deshmukh *et al*., 2023). With longer life expectancy in people living with HIV on effective antiretroviral therapy, the rate of anal cancer is likely to continue to rise (Piketty *et al*., 2012; Albuquerque *et al*., 2019). Current national HPV vaccination initiatives are likely to reduce HPV-related anal disease in the long term (Wang *et al*., 2022).

Oncogenic types of HPV infection, particularly HPV-16, are the primary cause of anal squamous cell carcinoma (Lin *et al*., 2018). Two systematic reviews published in 2012 and 2021 reported a pooled prevalence of anal HPV-16 infection among MSMLWH of 35.4% (Machalek *et al*., 2012) and 28.5% (Wei *et al*., 2021), respectively. Wei *et al*. (2021) found that nearly three-quarters of this population had at least one high-risk HPV type. A recent study conducted in France found a prevalence of 28.9% for HPV-16 and 41.1% for other high-risk HPV types among more than 500 MSMLWH aged 35 years or older (Clifford *et al*., 2018).

A recent meta-analysis reported MSMLWH to have the highest risk for anal cancer: average incidence 85 cases per 100 000 person-years (Clifford *et al*., 2021) in comparison with 1.8 in the general population (Smittenaar *et al*., 2016). No national data exist on anal cancer incidence by MSM or HIV status in the UK (Lin *et al*., 2017). However, estimates for MSMLWH in England indicate an incidence of 41.8 per 100 000 person-years among those aged 40–44 years that rises to 282.4 per 100 000 person-years among those aged 70 years or older (Lin *et al*., 2017). Survival is low among patients whose anal cancer is detected at an advanced stage (Wong *et al*., 2023), while standard chemoradiotherapy treatment may cause severe adverse effects and a reduction in health-related quality of life (Ludmir *et al*., 2017).

The pathogenesis of anal cancer includes a precancer phase characterised by anal intraepithelial neoplasia (AIN). The rationale for anal precancer screening is to detect high-grade squamous intraepithelial lesions (HSIL), the precursor of anal cancer, and refer patients for preventive treatment (Cruz *et al*., 2023).

Screening aims to detect cancer or precancerous lesions at a stage when treatment of cancer is most likely to be curative, or where treatment of precancer prevents the development of cancer (World Health Organization, 2017; Kim *et al*., 2022). Well-organised cervical cancer screening programmes have been highly effective in reducing the incidence of cervical cancer, providing a model for anal cancer screening among high-risk groups. MSMLWH diagnosed with anal cancer during screening have improved survival compared to those diagnosed when symptomatic (van der Zee *et al*., 2023). A recent HSIL treatment trial among 4446 individuals aged 35 years or older in the US found that treatment of anal HSIL in people living with HIV reduces the risk of anal cancer by 57% when compared with active surveillance (Palefsky *et al*., 2022). Recent global consensus-based guidelines recommend annual anal cancer screening for MSMLWH as the highest priority group (Stier *et al*., 2024; Albuquerque and Fontes, 2025), although national anal cancer screening programmes are yet to be implemented (Sanger *et al*., 2023).

Despite the growing interest in anal cancer screening in people living with HIV, little is known about the potential negative consequences of such interventions. Patient-reported outcome measures (PROMs) are quantitative measures of an individual’s health condition as experienced and reported by the patient. Developed through qualitative research with affected communities and literature review, PROMs evaluate specific constructs such as health-related quality of life (Kim *et al*., 2022). It is important to measure such outcomes so that the full impact of screening can be understood. For example, screening may consist of repeated examinations over time, potentially having a financial, time constraint, and psychological impact on the individual (Kim *et al*., 2022). Screening may lead to the incidental finding of other diseases and further management of these conditions (Bretthauer *et al*., 2023). Cancer diagnosis may result in negative psychological impacts such as stigma (Bretthauer *et al*., 2023). Further investigating false positive screening results has resource implications relating to time, cost, and inconvenience. Balancing such risks with the benefits of screening is crucial in developing successful cancer screening programmes.

HSIL treatment trials have rarely collected data on patient-reported outcomes (PROs), although core outcome sets that include PROs are currently in development (Finch, 2023). A health-related symptom index for individuals either treated or monitored for anal HSIL has been developed (Atkinson *et al*., 2023). Current screening tests for anal precancer and cancer include anal cytology (‘anal swab/smear’), digital anal-rectal examination (DARE), and high-resolution anoscopy (HRA) with directed biopsies. Varied national and international screening guidelines generally recommend a screening pathway of anal cytology with high-risk HPV testing followed by HRA referral in cases with abnormal or high-risk HPV results (Albuquerque and Fontes, 2025). Such procedures are not currently routine among MSMLWH and may be associated with physical and psychological discomfort. MSMLWH are unlikely to have previously experienced routine cancer screening, such as cervical or breast cancer screening, which means this is a novel intervention for this population. Living with a long-term condition, such as HIV, may increase anxiety about cancer risk and abnormal screening results (Knettel *et al*., 2021; Kim *et al*., 2022). Given the high prevalence of AIN among MSMLWH (Schofield *et al*., 2016), the population eligible for continued surveillance or further investigation if a triage test is used in a staged screening programme may be large, warranting further investigation of PROs.

In this paper, we reviewed the literature on PROs associated with anal precancer screening among MSMLWH and identified current research priorities. This is topical, as supported by the recent evidence detailed above, implementation of national screening programmes is likely to be imminent.

## Methods

This scoping review followed guidance for descriptive systematic scoping reviews (Arksey and O’Malley, 2005; Levac *et al*., 2010). The review addresses the following research question: What PROs are reported by MSMLWH who have participated in anal cancer or precancer screening?

### Identification of relevant studies

We systematically searched Medline, EMBASE, and APA PsycINFO databases in December 2024 for peer-reviewed journal articles, supplementing this with backwards and forwards citation searching. All study types were included. We used a predefined search strategy combining sets of terms relating to anal precancer (AIN)/cancer, screening, and PROs (see Supplementary Table S1, Supplemental digital content 1, https://links.lww.com/EJCP/A582 for full search strategy).

### Article selection

Search results were imported into EndNote (The EndNote Team, 2013), and duplicates were removed. The first and second authors (E.S. and Y.G.) independently screened all titles and abstracts. Articles selected for full-text review were uploaded into EndNote, and reviewed (by E.S. and Y.G.) to determine relevance and whether they fit the inclusion criteria. In the case of disagreement, E.S. and Y.G. reached consensus through discussion and review of the relevant paper, with the senior author (R.G.) providing a final decision if consensus could not be reached.

Inclusion criteria: Original studies published in English from 2000 to date (corresponding to the time when HRA became more widely used for the diagnosis of HSIL) (Goon *et al*., 2015) reporting PROs following screening for anal precancer or cancer (including after receiving results) among MSMLWH. Publications reporting on mixed populations, such as MSM living with and without HIV, were included if data on individuals living with HIV were reported separately.

Exclusion criteria: Publications not reporting separate outcomes for MSMLWH only.

### Charting the data

Data were extracted by E.S. and Y.G., using a standardised extraction sheet based on that used by Kim *et al*. (2022) (see Supplementary Table S2, Supplemental digital content 2, https://links.lww.com/EJCP/A583 for data extraction template). E.S. and Y.G. met to reach a consensus on the data.

## Results

**Fig. 1:**
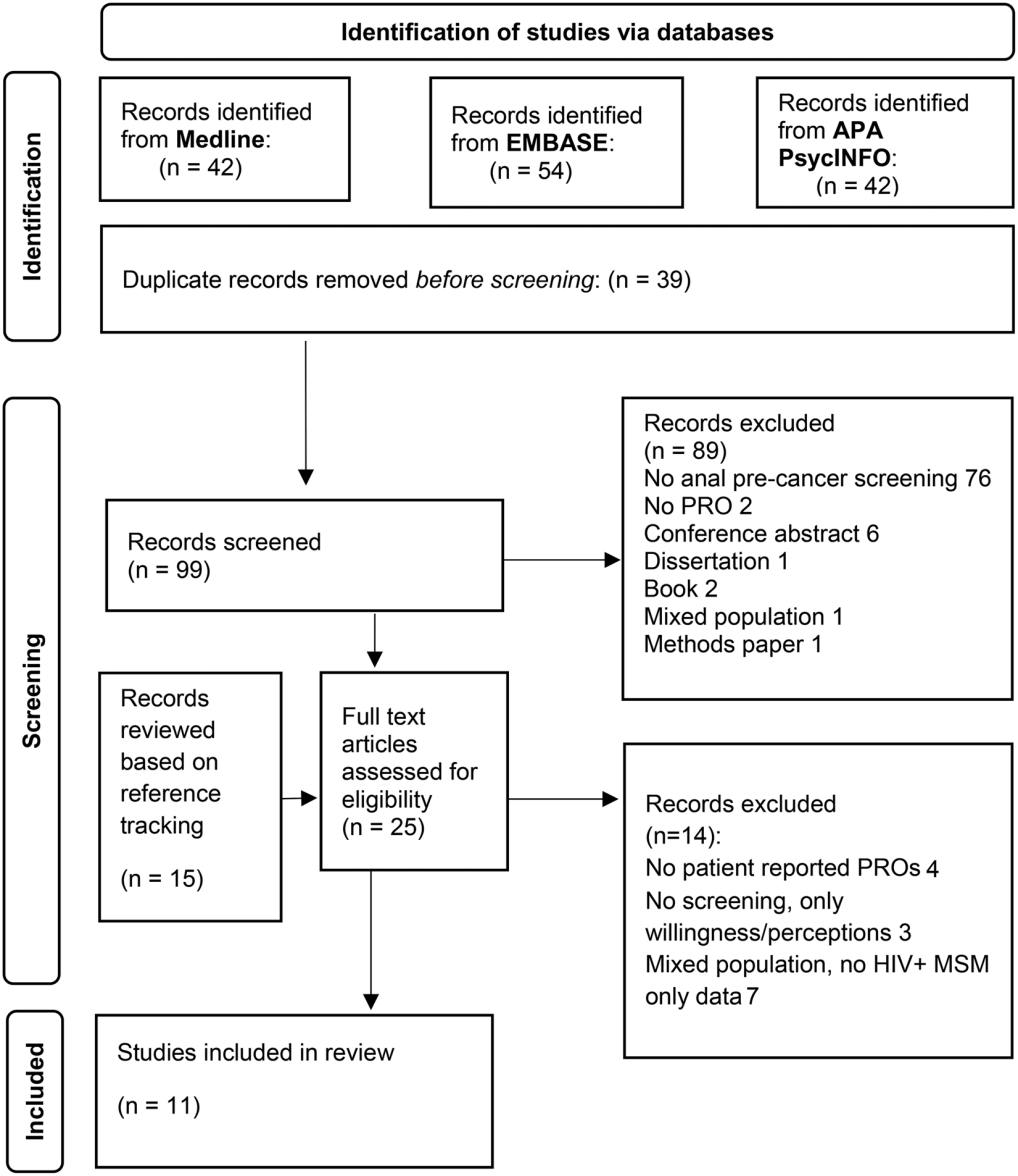
Flowchart showing the search results and screening process.

We identified 138 articles, with 99 remaining after deduplication (Fig. 1). We excluded 89 articles on title and abstract screening. A further 15 publications were identified through citation searching, resulting in 25 full-text articles being assessed for eligibility, of which 11 met our inclusion criteria. The 14 studies were excluded mainly because of a lack of separate reporting of participant groups by HIV status. Two papers were based on the anal cancer examination study (Ong *et al*., 2014) and two papers on the Study of the Prevention of Anal Cancer (SPANC) (Machalek *et al*., 2013). Given the heterogeneity of the 11 papers included, we report findings as a narrative synthesis, grouping studies by design and ordering by year of publication.

Study characteristics are presented in Table 1. Almost all papers reported on studies conducted in high-income settings (*n* = 10), including Australia (*n* = 5), Canada (*n* = 2), the US (*n* = 2), and the UK (*n* = 1). One article was based on a study from Nigeria. A majority of articles (*n* = 9) reported on quantitative studies, using validated or unvalidated instruments to measure PROs; almost half reported longitudinal data (*n* = 5). Timing of data collection ranged from immediately before the first screening test to after receiving histology results following the final screening test.

**Table 1:** Main characteristics of publications

Publication	Country	Aim	Participants and sample	Screening method	Data collection method	Assessment points	Patient-reported outcome domain
Longitudinal studies							
Ong *et al*. (2018)	Victoria, Australia	Assess patient uptake and experience of clinician-performed DARE	Individuals attending a sexual health centre, a tertiary hospital HIV outpatient clinic, or two high HIV case load general practices Sample size: 327 Mean age: 51 years (9 SD) Ethnicity: Born in Australia 69% Education level: Tertiary education 52% Mean years since HIV diagnosis: 13 (8 SD) Prior screening experience: Not reported	Clinician-performed annual DARE	Self-administered questionnaires (paper or online)	T1: Baseline survey T2: Year 1 DARE T3: Year 2 DARE	Health-related QoL Experiences of screening procedures
Read *et al*. (2013)	Melbourne, Australia	Assess acceptability of and variations in acceptability of a 3-monthly DARE over a year	A clinic-based cohort Sample size: 102 Median age: 48 years (range: 35–86 years) Ethnicity: Not reported Education level: Not reported Mean years since HIV diagnosis: Not reported Prior screening experience: Not reported	Clinician-performed DARE	Self-administered pen-and-paper and email questionnaires	T1 = Baseline, at recruitment after first DARE T2 = 2 weeks after second DARE T3 = 2 weeks after third DARE	Experiences of screening procedures
Landstra *et al*. (2013a)	Sydney, Australia	Examine the influence of DIDF and PF on men with HIV undergoing anal cancer screening	A clinic-based cohort of MSM living with HIV Sample size: 271 Mean age: 51 years (9 SD) Ethnicity: Not reported Education level: Tertiary education ~64% Mean years since HIV diagnosis: 15 (8 SD) Prior screening experience: Not reported	First self-collected anal cytology Second HRA if abnormal cytology results (*n* = 66; ~24%)	Self-administrated pen-and-paper questionnaires	T1 = Baseline, the day of self-collected anal cytology T2 = After histology results or matched time interval if no HRA (~12–14 weeks after the baseline)	Health-related QoL Psychological impact
Landstra *et al*. (2013b)	Sydney, Australia	Investigate the psychological impact of a naturalistic anal cancer screening programme	A clinic-based cohort of MSM living with HIV Sample size: 287 Mean age: 52 years (9 SD) Ethnicity: Not reported Education level: Tertiary education ~64% Mean years since HIV diagnosis: 15 (8 SD) Prior screening experience: 46% self-reported past anal HPV diagnosis at baseline	First self-collected anal cytology Second HRA if abnormal cytology results	Self-administered pen-and-paper questionnaires	T1 = Baseline, before self-collected anal cytology T2 = within a week of anal cytology results T3 = within a week of anal histology results or matched time internal if no HRA (control group)	Health-related QoL Psychological impact Physical health
Tinmouth *et al*. (2011)	Toronto, Canada	Measure the psychological impact, both positive and negative, of being screened for anal cancer	Individuals with a history of anal receptive intercourse who were attending tertiary care hospital HIV clinics or primary care clinics Sample size: 104 Median age: 44 years (IQR: 40–50 years) Ethnicity: Not reported Education level: Tertiary education 54% Median years since HIV diagnosis: 15 (IQR: 7–20) Prior screening experience: Not reported	1st anal cytology and HRA 2nd HRA at 6 months after the first visit	Self-administered questionnaires (paper or online)	T1: within a week before the initial visit T2: within a week after the initial visit T3: within a week after receiving results T4: within a week before 6 months’ follow-up visit	Health-related QoL Psychological impact Physical health
Cross-sectional studies							
Nowak *et al*. (2020)	Abuja, Nigeria	Assess satisfactionwith anal cancer screening using HRA among MSM living with or at risk for HIV	Individuals participating in an HIV treatment-as-prevention study Sample size: 178 PLWH Age in categories: ≤24 years 35.4% (63/178) 25–34 years 53.9% (96/178) ≥35 years 10.7% (19/178) Ethnicity: Not reported Education level: Not reported Mean years since HIV diagnosis: Not reported. 40% were diagnosed at study enrolment Prior screening experience: Not reported	Anal cytology, DARE and HRA	Self-administered questionnaire after HRA screening	T1 = after HRA screening (no further details of exact timing)	Psychological impact Experiences of screening procedures
Ong *et al*. (2016)	Victoria, Australia	Assess patient uptake and experience of clinician-performed DARE	Individuals attending a sexual health centre, a tertiary hospital HIV outpatient clinic, or 2 high HIV case load general practices Sample size: 327 Mean age: 51 years (9 SD) Ethnicity: Born in Australia 69% Education level: Tertiary education 52% Mean years since HIV diagnosis: 13 (8 SD) Prior screening experience: Not reported	Clinician-performed DARE	Self-administered questionnaires (paper or online)	T1: Baseline survey T2 : 2 weeks postbaseline DARE	Health-related QoL Psychological impact Experiences of screening procedures Physical health
Schofield *et al*. (2016)	Manchester, UK	Assess patient acceptability of screening for anal neoplasia	MSM attending sexual health clinics, some of whom were living with HIV Sample size: 203 HIV+ Median age: 42 years Ethnicity: White British 74.4% Education level: Not reported Mean years since HIV diagnosis: Not reported Prior screening experience: Not reported	First anal cytology, DARE and HRA Second HRA at 6 months to confirm initial results	Self-administered questionnaires	T1: First HRA visit T2: Second HRA visit at 6 months	Experiences of screening procedures
D’Souza *et al*. (2013)	USA	Examine acceptance, attitudes toward, experiences of and reasons to decline screening	Individuals participating in a prospective cohort study of HIV Sample size: 395 HIV+ Median age: Not reported for HIV+ only Ethnicity: Not reported for HIV+ only Education level: Not reported for HIV+ only Mean years since HIV diagnosis: Not reported Prior screening experience: 39% had previously been screened with 51% reporting an abnormal anal cytology	Anal cytology	A computer-assisted self-interview questionnaire	T1: 6 months before (52%) or just before (48%) anal cytology T2: ~6 months after anal cytology	Experiences of screening procedures
Qualitative studies							
Finneran *et al*. (2021)	Atlanta, US	Examine how a cohort of MSM living with HIV conceptualise anal cancer care, focussing on relationships between their understandings of gender and sexual orientation	Cisgendered MSM living with HIV Sample size: 15 Mean age: 51.2 years (range: 44–67) Ethnicity: Black/African-American 80% Education level: Not reported Mean years since HIV diagnosis: Not reported Prior screening experience: Not reported	Anal cytology and HRA	Four qualitative FGDs and enrolment survey	One FDG	Psychological impact
Gaspar *et al*. (2018)	Toronto, Canada	Better understand HIV-positive gay men’s perceptions of anal cancer and willingness to be screened and to examine how participants’ narrative accounts of living with HIV shaped their responses to anal cancer as a comorbidity risk	Cisgendered gay men living with HIV who participated in a clinical trial on anal cancer screening with varying anal cytology test diagnoses, treatment experiences, and primary care physicians Sample size: 25 Mean age: 50.4 years (9.99 SD) Ethnicity: White *n* = 20 Education level: Tertiary education *n* = 16 Mean years since HIV diagnosis: 15.1 (7.8 SD) Prior screening experience: First time screened	First anal cytology and DARE Second HRA if anal cytology abnormal Third those with HSIL received treatment or surveillance assigned random	In-depth interviews	A single interview at a varying point of the screening process; all had received their results	Psychological impact

DARE, digital anal rectal examination; DIDF, difficulty identifying and describing feelings; FDG, focus group discussion; HRA, high-resolution anoscopy; HSIL, high-grade squamous intraepithelial lesion; IQR interquartile range; MSM, men who have sex with men; PF, psychological flexibility; QoL, quality of life; T1, T2, T3, Data collection timepoints 1, 2, and 3.

In the majority of studies, participants were aged more than 40 years and had been diagnosed as living with HIV greater than or equal to 10 years previously. Ethnicity, educational attainment, and prior cancer screening experience were rarely reported. Where educational attainment was reported, between 52 and 69% had completed tertiary level education.

As the studies included utilised varied PROMs, we categorised the PRO constructs into meaningful domains of experience. In total, four domains were formed: (a) health-related quality of life (HRQoL), (b) psychological impact (including anxiety symptoms, depressive symptoms, distress, and worry), (c) experiences of screening procedure/s (including satisfaction with screening procedure/s, pain, comfort or discomfort, and embarrassment), and (d) physical health. The directions of impact on the PRO domain are presented in Table 2.

**Table 2:** Direction of impact on patient-reported outcome domain

Patient-reported outcome domain	PROM	Negative impact	No impact	Positive impact	Evidence of change over time
Health-related quality of life	SF-12 SF-6D IIRS	Tinmouth *et al*. (2011) Most psychological impact of chronic illness intrusiveness reported on at least 2 QoL items at T2 following HRA (IIRS)	Landstra *et al*. (2013a) **–** SF-12 PCS T1 : 49.60 (9.66 SD) T2 : 48.95 (9.70 SD) (anal cyt./HRA) Ong *et al*. (2016) **–** SF-6D T1 : 0.76 (0.13 SD) T2 : 0.77 (0.13 SD) (DARE) Landstra *et al*. (2013b) There were no significant differences between the groups (cyt. neg./LSIL vs. hist. neg./LSIL vs. hist. HSIL) at any time point for ratings of physical or mental health-related quality of life (*P* > 0.05) (anal cyt./HRA) Ong *et al*. (2018) **–** SF-6D T1 : 0.75 (0.13 SD) T2 : 0.76 (0.14 SD) T3 : 0.74 (0.13 SD) (*P* = 0.40) (DARE)		
Psychological impact					
* *Anxious symptoms	PCQ + DASS 21 Unvalidated questionnaire In-depth interview FDG	Nowak *et al*. (2020) **–** I was very anxious about having the procedure (HRA) Agree/strongly agree 91% Disagree/strongly disagree 4% Gaspar *et al*. (2018) **–** Anal cytology results caused anxiety relating to ageing with HIV (anal cyt./HRA) Finneran *et al*. (2021) **–** Lack of health literacy extended to HRA, and many participants spoke about not knowing what HRA entailed until their first HRA appointment, which created ‘fear and anxiety’	Landstra *et al*. (2013a) – DASS-21 T1 : 2.13 (3.36 SD) (i.e. ‘normal’) T2 : 2.59 (3.80 SD) (i.e. ‘normal’) (anal cyt./HRA) Landstra *et al*. (2013b) **–** There were no significant differences between the groups (cyt. neg./LSIL vs. hist. neg./LSIL vs. hist. HSIL) at any time point for ratings of anxiety (*P* > 0.05) (anal cyt./HRA)		Tinmouth *et al*. (2011) **–** Positive psychological impact of being screened scores were lowest after screening (HRA) and rose thereafter
Depressive symptoms	DASS 21		Landstra *et al*. (2013a) – DASS-21 T1 3.24 (4.34 SD) (i.e. ‘normal’) T2 3.55 (4.64 SD) (i.e. ‘normal’) (anal cyt./HRA) Landstra *et al*. (2013b) **–** There were no significant differences between the groups (cyt. neg./LSIL vs. hist. neg./LSIL vs. hist. HSIL) at any time point for ratings of depression (*P* > 0.05) (anal cyt./HRA)		
Distress	DASS-21 Unvalidated questionnaire In-depth interview	Ong *et al*. (2016) **–** T2: Life changed for the worse because of DARE results 2% (95% CI: 0–5) Gaspar *et al*. (2018) **–** Perceived ‘communal silence’ surrounding HPV-associated cancers caused distress at the time of receiving results (anal cyt./HRA) Landstra *et al*. (2013b) **–** T3: Hist. HSIL group experienced more distress than hist. neg./LSIL group *F*(1, 43) = 5.65, *P* = 0.02*, η*^2^ = 0.12 (HRA) Schofield *et al*. (2016) **–** Recalled distress while waiting to receive results **–** 4/203 reported scores ≥8 (0 = no distress, 10 = extreme distress) After receiving results **–** 5/203 with AIN results reported scores ≥8 (0 = no distress, 10 = extreme distress) (anal cyt./DARE/HRA)	Landstra *et al*. (2013a) **–** DASS 21 T1 4.37 (4.28 SD) (i.e. ‘normal’) T2 4.54 (4.53 SD) (i.e. ‘normal’) (anal cyt./HRA)		
Worry	Unvalidated questionnaire Adapted prostate cancer worry items from McNaughton-Collins *et al.* (2004) FDG	Ong *et al*. (2016) – T2: Worried about dying soon 4% (95% CI: 2–7) Worried about developing anal cancer 3% (95% CI: 2–7) (DARE) Landstra *et al*. (2013b) – T2: Participants referred to HRA reported higher levels of worry than no-HRA group T3: HSIL group continued to have higher levels of worry, whereas hist. neg./LSIL group dropped to between the HSIL and no-HRA group *F*(2159) = 16.59, *P* < 0.001, *η*^2^ = 0.17 (anal cyt./HRA) Gaspar *et al*. (2018) – Ambiguous anal cytology results were associated with increased anal cancer worry Finneran *et al*. (2021) – Participants spoke of fear or anxiety around rectal cancer as a new anxiety, and drew comparisons to past concerns about their prostate health (HRA)	Read *et al*. (2013) – Worried might not be clean during DARE T1: Not at all 42 (44%, 95% CI: 34–54) A little 37 (39%, 95% CI: 29–49) A fair bit/quite a lot/very much 17 (18%, 95% CI: 11–27) T2–T3: Not at all 52 (38%, 95% CI: 30–47) A little 61 (45%, 95% CI: 36–53) A fair bit/quite a lot/very much 24 (18%, 95% CI: 12–25)	Gaspar *et al*. (2018) – Successfully managing HIV was associated with reduced worry of HSIL diagnosis following HRA	
Experiences of screening					
Satisfaction	Unvalidated questionnaire IES	Ong *et al*. (2018) – Not feeling in control of their body during DARE at any timepoint in 14 of the 862 DAREs (1.6%, 95% CI: 0.9–2.7)	Read *et al*. (2013) – DARE examination was emotionally upsetting T1: Not at all 95 (99%, 95% CI: 94–100) A little/a fair bit 1 (1%, 95% CI: 0–6) T2–T3: Not at all 137 (100%, 95% CI: 97–100) A little A fair bit 0 (0%, 95% CI: 0–3) Read *et al*. (2013) – Overall experience of DARE T1: Unpleasant 4 (4%, 95% CI: 1–10) Neither good nor bad/acceptable 93 (96%, 95% CI: 90–99) T2–T3: Unpleasant 3 (2%, 95% CI: 0–6) Neither good nor bad/acceptable 134 (98%, 95% CI: 94–100)	Ong *et al*. (2016) – T2: Reassured about anal exam results following DARE 83% (95% CI: 78–88)	Schofield *et al*. (2016) – Willingness to be screened Every year 84/117 (71.8%) Every 2–3 years 27/117 (23.1%) Every 4–10 years 6/117 (5.2%)
Pain	Unvalidated questionnaire	Nowak *et al*. (2020) – I had a lot of pain during the procedure (HRA) Agree/strongly agree 11% Disagree/strongly disagree 49% Ong *et al*. (2016) – T2: Examination (DARE) caused pain 1% (95% CI: 0–3) Ong *et al*., (2018) – Anal pain during DARE at any timepoint was reported in 10 of 862 DAREs (1.2%, 95% CI: 0.6–2.1)	Read *et al*. (2013) – Pain during DARE examination T1: Little or none 97 (100%, 95% CI: 96–100) A fair bit/quite a lot 0 (0%, 95% CI: 0–4) T2–T3: Little or none 137 (100%, 95% CI: 97–100) A fair bit/quite a lot 0 (0%, 95% CI: 0–3) Schofield *et al*. (2016) Recalled pain Mean score during HRA: 3.7 (range: 0–9) Mean pain score after HRA: 3.5 (range: 1–10)		Schofield *et al*. (2016) – Post-HRA pain lasted an average of 3 days
Comfort/discomfort	Unvalidated questionnaire	Nowak *et al*. (2020) – The procedure (HRA) caused me great discomfort Agree/strongly agree 8% Disagree/strongly disagree 66% Ong *et al*. (2016) – T2 Found the DARE examination uncomfortable 5% (95% CI: 3–9) D’Souza *et al*. (2013) – T2 Found anal cytology uncomfortable 16% (65/395) and scary 4% (16/395)	D’Souza *et al*. (2013) – T2 found anal cytology ‘Not a big deal’ 82% (323/395) and ‘Not as bad as expected’ 64% (254/395)	Nowak *et al*. (2020) – The procedure (HRA) was more comfortable than I expected Agree/strongly agree 68% Disagree/strongly disagree 7%	
Embarrassment	Unvalidated questionnaire	Ong *et al*. (2016) – T2: Embarrassed by having anus touched by physician during DARE 8% (95% CI: 5–13)	Read *et al*. (2013) – Embarrassed having anus touched during DARE T1: Not at all/a little 92 (96%, 95% CI: 90–99) A fair bit/quite a lot 4 (4%, 95% CI: 1–10) T2–T3: Not at all/a little 137 (100, 95% CI: 97–100) A fair bit/quite a lot 0 (0, 95% CI: 0–3)		
Physical health	Unvalidated questionnaire Modified CSQ	Landstra *et al*. (2013b) – T2: Those referred to HRA rated their anal health worse than those who did not need HRA *F*(1157) = 14.22*, P* < 0.001, *η*^2^ = 0.08	Ong *et al*. (2016) – T2: Way body feels (better/same) 98% (95% CI: 96–100) General health (better/same) 100% (95% CI: 98–100) Anal health (better/same) 99% (95% CI: 97–100) Optimistic about future health (better/same) 98% (95% CI: 96–100)	Landstra *et al*. (2013b) – T2: No-HRA group were more optimistic about future health than those requiring HRA *F*(1158) = 8.40, *P* = 0.004, *η*^2^ = 0.05 Landstra *et al*. (2013b) – T3: No-HRA and HSIL groups rated their optimism about future health similarly. Hist. neg/LSIL group rated themselves most optimistic *F*(2158) = 3.19, *P* = 0.04, *η*^2^ = 0.04	

95% CI, 95% confidence interval; CSQ, Cervical Screening Questionnaire; DARE, digital anal rectal examination; DASS-21, Depression Anxiety Stress Scales; DIDF, difficulty identifying and describing feelings; FDG, focus group discussion; HRA, high-resolution anoscopy; HSIL, high-grade squamous intraepithelial lesion; IES, impact of event scale; IIRS, Illness Intrusiveness Ratings Scale; IQR, interquartile range; LSIL, low-grade squamous intraepithelial lesion; MSM, men who have sex with men; PCQ+, Prostate Cancer Worry Questionnaire; PF, psychological flexibility; PROM, patient-reported outcome measure; QoL, quality of life; SF-12, the Medical Outcomes Study Short Form Health Survey; SF-6D, Short-Form Six-Dimension; T1, T2, T3, data collection timepoints 1, 2, and 3.

### Health-related quality of life

HRQoL was reported in four papers using one of three validated PROMs: the Medical Outcomes Study Short Form Health Survey (SF-12) (Gandek *et al*., 1998), Short-Form Six-Dimension (Mukuria *et al.*, 2025), and Illness Intrusiveness Ratings Scale (IIRS) (Devins, 1994). There were no reported differences in HRQoL between people who had undertaken self-collected anal cytology and those who had undertaken self-collected anal cytology *and* subsequent HRA, regardless of results (Landstra *et al*., 2013b). In a longitudinal study of incorporating an annual DARE into routine HIV care, 327 participants undergoing annual DARE did not report any change in HRQoL (Ong *et al*., 2018). When measured longitudinally, the psychosocial impact of chronic condition on HRQoL (IIRS) was rated most negatively immediately after the screening HRA, ratings returning to prescreening levels when measured after receiving results (Tinmouth *et al*., 2011).

### Psychological impact

PROs related to psychological wellbeing were the most measured PRO among studies included in this review. Anxiety was assessed in studies reported in five papers using quantitative measures [*n* = 3, the validated Depression, Anxiety and Stress Scale-21 (Lovibond and Lovibond, 1995) and an unvalidated questionnaire] and qualitative approaches (*n* = 2, in-depth interviews and focus group discussions). In an HIV treatment-as-prevention study which explored satisfaction with HRA for anal cancer screening, the majority of MSMLWH (91%) expressed anxiety about undergoing HRA (Nowak *et al*., 2020); however, in two papers from the SPANC study, self-reported anxiety (DASS-21) did not differ between baseline and after receiving results for self-collected anal cytology or histology results following HRA (Landstra *et al*., 2013a, 2013b). The two qualitative studies described how anxiety was largely related to limited knowledge of screening procedures and difficulties understanding screening results (Gaspar *et al*., 2018; Finneran *et al*., 2021).

Only one study assessed depressive symptoms, finding no significant difference between baseline and later in the screening process, regardless of results (Landstra *et al*., 2013a, 2013b).

Distress was referred to in five papers reporting on four studies using DASS-21 (*n* = 2), an unvalidated questionnaire (*n* = 2), and in-depth interviews (*n* = 1). Reported distress scores were similar at baseline and after receiving histology results following HRA (Landstra *et al*., 2013a). Yet, those who were referred for HRA following abnormal self-collected anal cytology, and who subsequently had HSIL on histology, experienced more distress than those whose histology results were normal or showed LSIL (Landstra *et al*., 2013b). A small number (<3%) of participants in a prospective questionnaire study of the feasibility and acceptability of anal screening in MSM in the UK reported distress while waiting for screening results or when receiving screening-positive results following HRA (Schofield *et al*., 2016). In an Australian study of annual DARE in routine HIV care, a few participants (2%) reported their life changing for the worse because of their screening results at 2 weeks post-DARE (Ong *et al*., 2016). In a qualitative interview study of patient experiences of anal cancer screening, lack of discussion of anal cancer, ‘communal silence’ among MSMLWH in Canada was perceived to contribute to distress at the time of receiving screening results following HRA (Gaspar *et al*., 2018, p.286).

### Worry

Five papers assessed worry specifically related to anal precancer or cancer screening using one of the following: a modified version of the Prostate Cancer Worry Questionnaire (*n* = 1) (Roth *et al*., 2003), an unvalidated questionnaire (*n* = 2), in-depth interviews (*n* = 1), and focus group discussions (*n* = 1). Nearly one-fifth of participants (18%) reported feeling worried they might not be clean during screening; this did not change over a 12-month period of 3-monthly clinician-performed DARE (Read *et al*., 2013). In another longitudinal study of DARE in routine HIV care, a minority of patients reported worrying about anal cancer or dying soon, in the 2 weeks after clinician-performed DARE (3 and 4%, respectively) (Ong *et al*., 2016). At the time of receiving anal cytology results, those referred for HRA reported higher levels of cancer worry than those who were not referred (Landstra *et al*., 2013b). Individuals with HSIL continued to have higher levels of worry focussed on anal precancer screening, whereas those whose histology was normal or showed LSIL had worry levels intermediate between those with HSIL and the no-HRA groups (Landstra *et al*., 2013b). In a qualitative study, previous experience of cancer increased anal cancer worry among MSMLWH (Finneran *et al*., 2021). The participants reported that the experience of living with HIV, another long-term condition, might have reduced their level of concern about anal cancer(Gaspar *et al*., 2018).

When assessed using the Psychological Consequences Questionnaire positive impact of screening was lowest immediately after screening test/s and rose thereafter (Tinmouth *et al*., 2011).

### Experiences of screening procedures

Experiences of the screening procedures were reported in six articles, all using unvalidated questionnaires to assess PROs related to this domain with one article relying on a single question of patient experience of anal cytology (D’Souza *et al*., 2013). An overwhelming majority of participants did not find clinician-performed DARE ‘unpleasant’, with no change over 12 months of 3-monthly DAREs (Read *et al*., 2013). Individuals reported minimal pain (1%), almost all (98%) felt ‘in control’ of their body during DARE (Ong *et al*., 2018), and the proportion of participants reporting embarrassment during DARE reduced from 4% during the first DARE to none during subsequent DAREs (Read *et al*., 2013). A majority (82%) of patients found anal cytology ‘Not to be a big deal’ while some (16%) found it uncomfortable or scary (4%) (D’Souza *et al*., 2013). Nowak *et al.* (2020) found that 11% of study participants experienced pain during HRA; however the majority (68%) found the procedure less uncomfortable than anticipated (Nowak *et al*., 2020). Authors reported no correlation between the number of biopsies and pain scores (Nowak *et al*., 2020). Duration of pain following HRA was a mean of 3 days (Schofield *et al*., 2016). A majority (>70%) of participants in a prospective anal cancer screening study among British MSM reported willingness to be screened annually (Schofield *et al*., 2016). Yet, when interviewed, MSMLWH found that the perceived female-gendered language associated with HPV and anal cancer screening further challenged their identities as sexual minority males (Finneran *et al*., 2021).

### Physical health

Anal health and broader general health were assessed in two studies (Landstra *et al*., 2013b; Ong *et al*., 2016) using a modified Cervical Screening Questionnaire (Wardle *et al*., 1995) and an unvalidated questionnaire, respectively. Referral to HRA following abnormal self-collected anal cytology results impacted ratings of anal health negatively (Landstra *et al*., 2013b). Participants who did not require HRA were more optimistic about their future general health than those referred for HRA (Landstra *et al*., 2013b). Undergoing DARE did not impact perceptions of general or anal health (Ong *et al*., 2016).

## Discussion

In this scoping review of literature on PROs associated with anal precancer screening among MSMLWH we found that screening is well tolerated with little impact on psychological wellbeing. A small proportion of individuals experienced high levels of distress related to the screening procedures. The modest number of relevant studies demonstrates the lack of a systematic assessment of PROs following anal precancer or cancer screening among MSMLWH.

The studies we identified showed that the screening process did not significantly affect participants’ overall psychological wellbeing. Anal precancer screening studies reporting on varied patient populations have produced similar findings. A qualitative study among Spanish men and women living with HIV undergoing anal precancer screening using HRA found that while many reported experiencing fear at some point during the screening process, previous experience of the tests promoted willingness to undergo screening again (Diez-Martinez *et al*., 2024). Similarly, another study of anal self-examination with more than 90% MSM of whom less than half were living with HIV, reported some discomfort in discussing anal health with their clinician but experienced no excess anxiety relating to anal cancer screening (Flores *et al.*, 2024).

It is important to note that all the papers involved screening as part of a study. Individuals who volunteer for research may be more health-conscious than those who decline participation (Jordan *et al*., 2013). Although those screened outside a research setting also tend to be more health-conscious than those who decline (Dryden *et al*., 2012). Increased health consciousness may buffer against the negative psychosocial impacts of screening. A study assessing anal precancer screening programme implementation in Australia reported a high nonparticipation rate (73.5%) among service users living with HIV (Dyer *et al*., 2025a), suggesting that, at least in a research context, clinic-based screening may not be acceptable to all. An RCT of community-recruited asymptomatic cisgender sexual minority men of whom nearly a third (27.1%) were living with HIV, reported higher engagement in home-based screening using anal cytology compared with clinic-based screening (Nyitray *et al*., 2023); a trend that persisted during annual screening (Nitkowski *et al*., 2025). Overall, among the participants living with HIV, home swabbing was highly acceptable and did not impact future HRA uptake in clinic (Nitkowski *et al*., 2024a, 2024b), suggesting that, in addition to the screening method, context is important. High acceptability of home swabbing is in line with findings in cervical cancer screening (Bishop *et al*., 2019), in which home swabbing has increased screening coverage (Serrano *et al*., 2022).

We found that screening impacted participants’ quality of life ratings immediately after the procedure (Tinmouth *et al*., 2011). Participants also felt least optimistic about their future health when receiving screening results (Landstra *et al*., 2013b). Similarly, a mixed population of MSM living with and without HIV who received abnormal screening results reported poorer HRQoL at 2 weeks after screening, but the decrease in QoL did not persist at three months (Cvejic *et al*., 2020). It is not surprising that anal precancer screening procedures impact psychosocial health immediately after the screening procedure, given that bowel function is a significant factor in patients’ quality of life ratings (De *et al*., 2021). Positive screening experiences, in particular in relation to discomfort, pain and low anxiety, are important in enhancing retention (Kutner *et al*., 2024).

As a further indication of tolerability, in the studies we reviewed the reported levels of pain or embarrassment were modest. Nowak *et al*. (2020) observed the highest proportion of participants reporting pain following HRA, at around 11%. Low pain is a positive finding, as a higher self-perceived risk for anal cancer and positive beliefs regarding screening (such as low pain) have been linked to a greater willingness to undergo screening (Geba *et al*., 2024). Consistent with our findings, previous studies among diverse populations recommended for or undergoing anal precancer screening have reported that fear of pain or embarrassment were not prominent barriers in screening attendance (Cruz *et al*., 2023).

The modest impact on general psychological wellbeing observed in many studies could be influenced by several factors. Many patients underwent either DARE with immediate results, or self or clinician-performed anal cytology only, with relatively few being referred for the more invasive HRA procedure. Studies of MSM living with and without HIV have reported high screening acceptability when using either clinician or self-collected anal swab (Dyer *et al*., 2025c), although acceptability is not always reported (Dyer *et al*., 2025b). In addition, the small sample sizes in the studies we reviewed may have limited the ability to detect statistically significant differences. Finally, prior screening experience, although often unreported, tends to reduce anxiety related to screening (Cruz *et al*., 2023).

In a small proportion of participants, the screening process increased their cancer-related worry, particularly among those requiring further examination. This aligns with previous findings. A cross-sectional questionnaire study among Puerto Rican men and women living with HIV found that those who expressed concern about developing anal cancer were more likely to have undergone anal cytology or HRA (Cruz *et al*., 2023). In this review, we found that those living with HIV may be proactive in managing anal cancer risk (i.e. seek screening) but may also experience stress related to the screening process. For some, even receiving negative screening results was emotionally challenging, as it evoked memories of their HIV diagnosis (Finneran *et al*., 2021). Those with more HIV-related symptoms were more likely to be anxious about screening (Tinmouth *et al*., 2011). Landstra *et al*. (2013b) presented a contrary view when they hypothesised that people living with HIV might be less concerned about anal precancer screening, as they are accustomed to frequent health checks associated with managing their chronic condition. For these individuals, the distressing aspects of screening may appear minor compared with the challenges posed by their HIV diagnosis and treatment. Gaspar *et al*. (2018) provide partial support for this hypothesis, as their interviewees compared the perceived risk of anal cancer to their experience of successfully managing their chronic condition. Future systematic assessment of PROs following anal precancer screening among MSMLWH would help us to answer this question.

## Implications (clinical and research)

Findings from this scoping review can inform clinical practice and future research. Distress related to procedures and limited understanding of screening results underscores the need for clear and informative communication in healthcare settings. By discussing the procedures with individuals in advance, healthcare providers not only give individuals the opportunity to learn about their future examination and ask questions, but to also identify those who may be particularly anxious about the screening. As cancer screening is a long-term process in which patients undergo tests and receive results that inform future screening decisions, patient experiences and the need for support may change over time (Tinmouth *et al*., 2011).

There is a paucity of literature on PROs associated with anal precancer screening among MSMLWH, as well as a lack of consistency in measuring PROs when this is undertaken. Our review highlights the need to incorporate standardised PROMs when evaluating and implementing anal precancer screening. Such tools allow us to measure unintended consequences of screening and will enhance the comparability of results. This is especially important as national screening efforts are expected to increase with the publication of the global consensus-based screening guidelines for this population (Stier *et al*., 2024).

There are limitations to this scoping review. We only identified 11 eligible studies, which were varied in design. Heterogeneity of the studies precluded meta-analysis. Sociodemographic characteristics such as ethnicity were rarely reported, so we are unable to investigate some potential health inequalities related to screening. Finally, all PROs were measured in participants taking part in studies on precancer or cancer screening. By recruiting from clinical studies, rather than routine clinical care the populations included may have been biased to those with more active health-seeking behaviours and a different perception of the screening experience. Therefore, our findings may not be directly applicable to routine clinical screening. A methodological limitation of this review was that we included only English-language papers and did not search grey literature, which may have introduced an information bias. Some publications were excluded as they did not report data for MSMLWH separately.

### Conclusion

In this scoping review of literature on PROs associated with anal precancer screening among MSMLWH, we found that systematic PROs after anal precancer screening among MSMLWH are rarely collected. But when reported, anal precancer screening was well tolerated with no impact on general psychological wellbeing. A very small proportion of individuals experienced high levels of distress related to the screening procedures. Assessing PROs may facilitate targeted support during screening to avoid adverse psychological consequences of screening.

## Acknowledgements

This study received grant support from the Central and North West London NHS Foundation Trust Research Starter Grant 2023.

### Conflicts of interest

There are no conflicts of interest.

## Supplementary Material
