# Supplementary material for: Impact of anal precancer screening on patient-reported outcomes among men-who-have-sex-with-men living with HIV: a scoping review
Source: Eur J Cancer Prev. 2025 Dec 17;35(5):473–85. doi: 10.1097/CEJ.0000000000001001 (PMC13412347; doi:10.1097/CEJ.0000000000001001)
Supplement: Supplementary file 1 [file ejcp-35-473-s001.docx]

**Supplementary Table 1 Search Strategy**

| **#** | **String** |
| --- | --- |
|  | **January 2000 to December 2024, English only**  **Search executed: 10/12/2024** |
| 1 | exp Anal Canal/ or exp Carcinoma, Squamous Cell/ or exp Anus Neoplasms/ |
| 2 | anal canal or anal cancer or rectal neoplasm or anal intraepithelial neoplasia or AIN or anal squamous cell carcinoma or squamous intraepithelial precancerous lesion* or high-grade squamous intraepithelial lesion* or high grade squamous intraepithelial lesion* or HSIL or anal dysplasia or anal or anus cancer or human papillomavirus or HPV **limited to abstracts** |
| 3 | anal canal or anal cancer or rectal neoplasm or anal intraepithelial neoplasia or AIN or anal squamous cell carcinoma or squamous intraepithelial precancerous lesion* or high-grade squamous intraepithelial lesion* or high grade squamous intraepithelial lesion* or HSIL or anal dysplasia or anal or anus cancer or human papillomavirus or HPV **limited to title** |
| 4 | #1 OR #2 OR #3 |
| 5 | exp "Early Detection of Cancer"/ |
| 6 | cancer screening or digital ano-rectal examination or digital anorectal examination or digital ano rectal examination or DARE or tertiary screening or high resolution anoscopy or high-resolution anoscopy or HRA or anoscopy or Mass screening or oncology or early detection of cancer or anal smear or anal cytology or anal pap or cancer screening test or screening **limited to abstracts** |
| 7 | cancer screening or digital ano-rectal examination or digital anorectal examination or digital ano rectal examination or DARE or tertiary screening or high resolution anoscopy or high-resolution anoscopy or HRA or anoscopy or Mass screening or oncology or early detection of cancer or anal smear or anal cytology or anal pap or cancer screening test or screening **limited to title** |
| 8 | #5 OR #6 OR #7 |
| 9 | exp "Sexual and Gender Minorities"/ or exp Homosexuality, Male/ |
| 10 | MSM or men who have sex with men or gay or men-who-have-sex-with-men or "Sexual and Gender Minorities" or homosexuality **limited to abstracts** |
| 11 | MSM or men who have sex with men or gay or men-who-have-sex-with-men or "Sexual and Gender Minorities" or homosexuality **limited to title** |
| 12 | #9 OR #10 OR #11 |
| 13 | exp Patient Reported Outcome Measures/ |
| 14 | Patient-reported outcome* or Patient reported outcome* or PROM or PROMs or PRO or PROs or questionnaire or survey or in-depth or interview or semi-structured or semi structured **limited to abstracts** |
| 15 | Patient-reported outcome* or Patient reported outcome* or PROM or PROMs or PRO or PROs or questionnaire or survey or in-depth or interview or semi-structured or semi structured **limited to title** |
| 16 | #13 OR #14 OR #15 |
| 17 | exp Stress, Psychological/ or exp Mental Disorders/ or exp Depression/ or exp "Quality of Life"/ or exp Distress, Psychological/ |
| 18 | Psychological stress or Psychological symptoms or Psychological distress or Psychological well-being or Psychological wellbeing or Psychological impact or functional status or health-related quality of life or HRQL or HRQoL or psychosocial status or psychosocial symptoms or psychosocial distress or psychosocial well-being or psychosocial wellbeing or psychosocial impact or anxiety or distress or worr* or fear or quality of life or QoL or quality of wellbeing or quality of well-being or disability or patient satisfaction or patient experience* or emotion* or depress* **limited to abstracts** |
| 19 | Psychological stress or Psychological symptoms or Psychological distress or Psychological well-being or Psychological wellbeing or Psychological impact or functional status or health-related quality of life or HRQL or HRQoL or psychosocial status or psychosocial symptoms or psychosocial distress or psychosocial well-being or psychosocial wellbeing or psychosocial impact or anxiety or distress or worr* or fear or quality of life or QoL or quality of wellbeing or quality of well-being or disability or patient satisfaction or patient experience* or emotion* or depress* **limited to abstracts** |
| 20 | #17 OR #18 OR #19 |
| 21 | #4 AND #8 AND #12 AND #16 AND #20 |
| 22 | limit 21 to (english language and yr="2000 -Current") |
|  | **Anal cancer AND screening AND MSM AND Proms AND psychological impact** |
|  |  |
|  |  |
|  |  |
|  |  |
|  |  |
|  |  |
|  |  |
|  |  |
|  |  |
|  |  |
|  |  |
|  |  |
|  |  |
|  |  |
|  |  |
|  |  |
|  |  |
